# Nutritional Intervention Improves Muscle Mass and Physical Performance in the Elderly in the Community: A Systematic Review and Meta-Analysis

**DOI:** 10.3390/life14010070

**Published:** 2023-12-31

**Authors:** Yuanyuan Ren, Aming Lu, Bingqing Wang, Cenyi Wang

**Affiliations:** School of Physical Education and Sports Science, Soochow University, Suzhou 215006, China

**Keywords:** nutritional intervention, muscle mass, physical performance, elderly, community

## Abstract

Nutritional supplements have been extensively used as health interventions for the elderly. However, with the spread of COVID-19, no consensus exists on whether nutritional interventions could improve muscle mass and physical activity in community-dwelling older adults. To conduct a systematic review and meta-analysis to explore the effects of different nutritional interventions on muscle mass and physical performance in the elderly, we searched *PubMed*, *Web of Science*, *Elsevier*, and *Cochrane* databases from their founding dates to December 2023. The meta-analysis was performed using RevMan5.3 software. Only randomized controlled trials (RCTs) were considered, and the overall mean difference (MD) or standardized mean difference (SMD) with 95% confidence interval (CI) was calculated. There were 33 studies comprising 3579 elderly persons meeting the inclusion criteria. Comprehensive analysis suggested that the intervention effect of fat-free mass (FFM), appendix skeletal muscle mass (ASMM), handgrip strength (HGS), gait speed, and short physical performance battery (SPPB) score was higher in the nutritional supplement group than in the control group. The results of subgroup analysis demonstrated that protein supplementation (SMD = 0.82, *p* < 0.0001) had an optimal effect on ASMM (SMD = 0.89, *p* < 0.0001) and FFM (MD = 2.09, *p* < 0.0001) in the elderly. Vitamin D supplementation (SMD = 0.52, *p* < 0.0001) had a marginal effect on ASMM, and energy supplementation (SMD = 0.39, *p* = 0.0005) had the lowest effect. Moreover, nutritional interventions had the most significant impact on HGS (MD = 1.06, *p* < 0.0001) and TUG (MD = 0.14, *p* < 0.0001) in individuals aged 65–75 years old, with positive effects on FFM (MD = 1.62, *p* < 0.0001) and HGS (MD = 0.82, *p* < 0.0001) when compared to healthy elderly individuals, and had greater effect on ASMM (SMD = 0.69, *p* < 0.0001) than on the elderly with sarcopenia. Nutritional supplements can enhance muscle mass and physical performance in the elderly, while protein is recommended for muscle function. The golden period for implementing nutritional interventions to improve muscle function is before the age of 75 years. However, the impact of nutritional interventions varies with age and population. Given the limited evidence on nutritional interventions, more detailed and high-quality studies are highly warranted in the future.

## 1. Introduction

With increasing age, physical function declines, resulting in the loss of independence and increased risk of falls. Malnutrition, weight loss, muscle strength weakening, and immune dysfunction are considered manifestations of aging. In particular, the attenuation of muscle function is deemed a crucial clinical marker of biological aging. Previous studies have reported that age-related decreases in skeletal muscle mass and function could lead to fractures and increase the risk of falls and fall-related injuries. Moreover, the loss of skeletal muscle function can result in diseases such as sarcopenia, which can considerably increase the medical burden of the elderly and raise their hospitalization and mortality rates [1].

Recently, there have been increasing studies on the health benefits of nutritional supplement interventions in the elderly, and the intake of a series of nutrients, such as energy, protein, lipids, and vitamins, can delay the development of frailty in the elderly to a certain extent [2]. One study pointed out that macronutrient and micronutrient supplementation and the maintenance of healthy nutrition can reduce the risk of frailty in the elderly [3]. Another study also documented that appropriate vitamin or mineral intake can significantly prevent or reduce depressive symptoms in the elderly [4]. Other randomized controlled trials (RCTs) linked to physical exercise have demonstrated that the combination of nutrition and physical exercise has an affirmative influence on cancer patients with metastatic or locally advanced gastrointestinal tract and lung tumors [5]. It has been widely accepted that a proper diet, balanced nutrition, and exercise could delay aging, alleviate depression, and reduce the risk of diseases and death in the elderly. However, with the spread of COVID-19, nutritional intervention for the elderly in the community has attracted increasing attention. Numerous studies have evinced that different types of nutritional supplements used for nutritional intervention or combined treatment for community-dwelling frail older adults can significantly improve the indicators of knee joint strength, physical activity, and walking speed [6]. Some researchers from Japan [7], South Korea [8], and the Netherlands [9] have also described the health-promoting effect of nutritional intervention on the elderly in the community. Likewise, China is constantly exploring nutritional intervention on community platforms. For example, one study administered a whey protein oral nutritional supplement to a group of older adults for 12 weeks and observed that whey protein nutritional supplement combined with resistance exercise positively influenced skeletal muscle function in the frail elderly compared to resistance exercise [10]. In another instance, nutritional and non-nutritional supplementary interventions were performed on Chinese elderly with sarcopenia living in a community, and significant improvements were observed in the outcomes of lower-limb muscle mass and five-chair stand test in the elderly in the nutritional intervention group [11]. Other researchers studied the frail elderly population using nutritional supplements or education. They noted that protein supplements alone for three months improved the physical function of the frail and malnourished elderly following hospitalization [12]. A study also carried out nutritional intervention with simultaneous energy and protein supplements in obese and functionally restricted elderly individuals for 6 months, thereby improving the quantity and quality of protein intake in the intervention group. The study’s results revealed that the physical function in the intervention group was significantly higher than that of the control group [13].

Although these interventional projects occupy a pivotal position, the absence of a detailed summary of nutrition intervention methods at the beginning of the study design leads to contradictory results. As the results of a recent meta-analysis demonstrated, the administration of nutritional supplements alone might be ineffective in frail elderly patients. In contrast, a combination of physical exercise and nutritional intervention could significantly improve various parameters, such as muscle strength, physical activity, and health-related quality of life in the elderly. Another cross-sectional study estimated the correlation between dietary protein intake, ASMM, and muscle mass (MM) by multivariable linear regression analyses. The results validated that ASMM and MM were significantly correlated with dietary animal protein intake in Japanese women aged 75 years or older but not with dietary plant protein intake. In contrast, dietary protein intake was not associated with ASMM or MM in men aged 65 or older. However, a meta-analysis found that high plant protein intake did not lead to a high skeletal muscle index, and plant-based protein might be negatively associated with the prevalence of sarcopenia [14]. This situation indicated that dietary protein and other nutritional intakes might have distinct effects on muscle function in older people of different ages and genders [15]. Moreover, Helio et al. [16] explored the relationship between protein intake and physical function in older adults and found that a protein intake higher than the recommended dietary allowance is cross-sectionally associated with better physical performance and greater muscle strength. Further studies have corroborated that the nutritional and functional statuses of the elderly living in communities differ from those living in long-term care institutions [17], and the interventional effects of nutritional support on the elderly in diverse environments are inconsistent. Although nutritional interventions and physical activity have been shown to decrease the risk of muscle function decline in the elderly, various factors in the study might have contributed to different findings, including nutritional supplements (type of nutrition), population characteristics (age, gender, and ethnicity), physical status (healthy, frail, sarcopenia, etc.), living environment (community, inpatient, nursing home, etc.), and muscle quality assessment methods. Therefore, a systematic review and meta-analysis of nutritional interventions for the elderly in communities were conducted in order to understand the current application of nutritional intervention methods and the response to the intervention as well as to explore the potential challenges in nutritional interventions so as to provide a scientific theoretical basis for the optimization of the selection of nutritional supplements for community-dwelling elderly populations.

## 2. Methods

### 2.1. Search Strategy

Pieces of literature were searched on *Cumulative Index to Nursing and Allied Health* (*CINAHL*), *Cochrane Library*, *EMBASE*, *PubMed*, *Scopus*, *SPORT Discus*, and *Web of Science* with the keywords “nutrition”, “supplements”, “diet”, “nutraceutical”, “protein”, “creatine”, “older adults”, “elderly”, “aging”, “community”, “community-dwelling”, “muscle mass”, “muscle strength”, “exercise”, and “ physical activity”, following the PICOS (Population, Intervention, Comparison, Outcome, Study) design format, through December 2023, and the search scope was limited to English.

### 2.2. Inclusion and Exclusion Criteria

Inclusion criteria included the following: (1) Participants living in the community were aged 65 years or older. (2) Randomized controlled studies were eligible for inclusion, and the experimental groups included single or multiple-factor nutritional interventions, such as a nutritional intervention group compared to a control group or physical activity combined with nutritional intervention when compared to physical activity alone. There was no significant difference in the outcomes between the intervention and control groups before the experimental intervention. (3) Outcomes included muscle mass, muscle strength, physical performance, etc. One or more of these outcomes were eligible for inclusion in this study.

Exclusion criteria included the following: (1) studies without focus on the elderly or older patients with cancer, neurodegenerative diseases, and depression; (2) reviews and systematic review articles or experimental studies (RCTs and non-RCTs) without nutritional intervention; and (3) experimental studies with outcomes not meeting the inclusion criteria.

### 2.3. Research Selection and Data Extraction

Two authors (Wang and Ren) initially selected research papers according to the titles and abstracts, and the papers meeting the inclusion criteria for full-text reading and targeted evaluation were chosen. All steps were carried out independently by two investigators, and a third investigator (Chen) resolved disagreements to reach a consensus when the first two authors disagreed or could not reach a decision. The extracted data included author, year of publication, country, baseline demographics of subjects (number, age, gender, and country), nutritional intervention (supplement type, dose, time, and frequency), skeletal muscle-related parameters (muscle mass, strength, and physical performance), and other results.

### 2.4. Literature Quality Assessment

The “Risk of Bias tool” in the Cochrane Manual was utilized to assess the risk of bias and the quality of the articles, mainly from six aspects: random sequence generation, allocation concealment, blinding of participants and personnel, blinding of outcome assessment, incomplete outcome data, and selective reporting. Moreover, the certainty of the evidence was assessed using the Grading of Recommendations, Assessment, Development and Evaluations (GRADE) approach. The evidence profile was created using GRADEpro GDT (GRADEpro Guideline Development Tool (Software), https://methods.cochrane.org/gradeing/gradepro-gdt, access on 25 September 2023).

### 2.5. Statistical Analysis

The fixed-effect or random-effect model was used for statistical analysis, and the MD or SMD was determined (95% CI). Our study judged the heterogeneity among the results with *I*^2^, and the higher the *I*^2^, the greater the degree of heterogeneity between studies. According to the Cochrane standard, the fixed-effect model was adopted if the heterogeneity among the results was small (*I*^2^ < 50%); otherwise, the random-effect model (*I*^2^ > 50%) was adopted, and subgroup analysis and sensitivity analysis were performed to test the reliability of the results. A funnel plot was used to evaluate publication bias in the study, and α = 0.05 was considered statistically significant.

## 3. Results

### 3.1. Literature Search Results and Basic Characteristics

**Figure 1:**
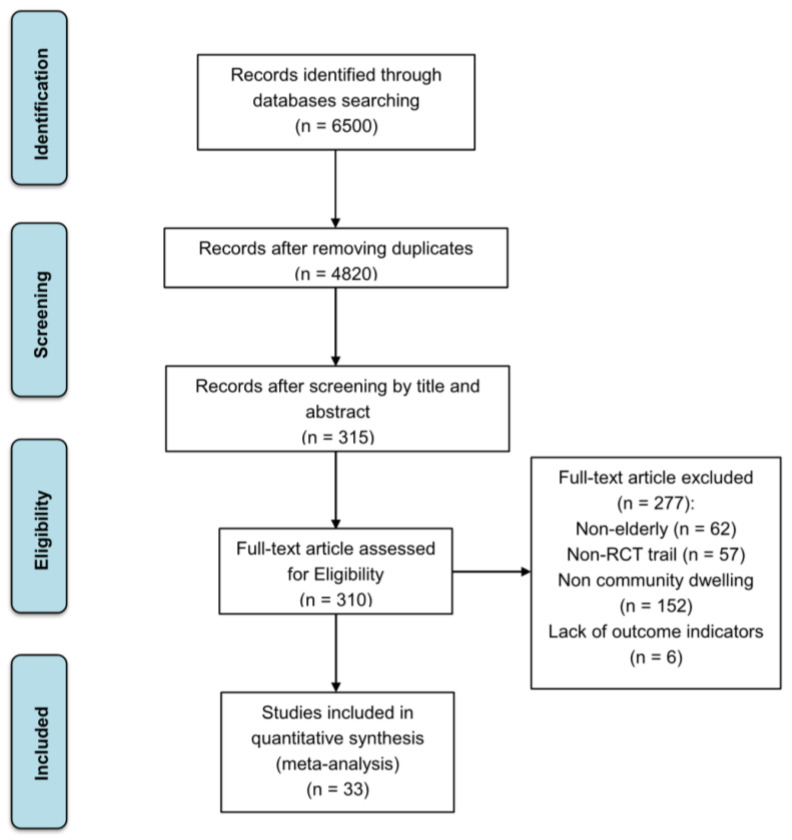
Flow diagram of included and excluded studies.

In total, 6500 articles were retrieved. The documents were initially screened by reading of the titles and abstracts, and the selected articles were further manually reviewed. Finally, 33 articles were included in this study (Figure 1).

Among the eligible studies, 19 were from Asia (8 from Japan, 3 from Republic of Korea, 3 from Singapore, 3 from China, 1 from the Philippines, and 1 from India), 10 were from Europe (4 from The Netherlands, 2 from Sweden, 1 from Norway, 1 from Finland, 1 from Hungary, and 1 from the UK), 3 were from North America (Canada, Winston Salem, and New Mexico), and 1 was from Oceania (Australia) (Table 1).

### 3.2. Literature Quality and Publication Bias Risk

“High risk”, “low risk”, and “unclear” were used to determine each index in the included literature, and the results are outlined in Table 2.

The certainty of evidence for each outcome based on GRADE is presented in Table 3.

**Figure 2:**
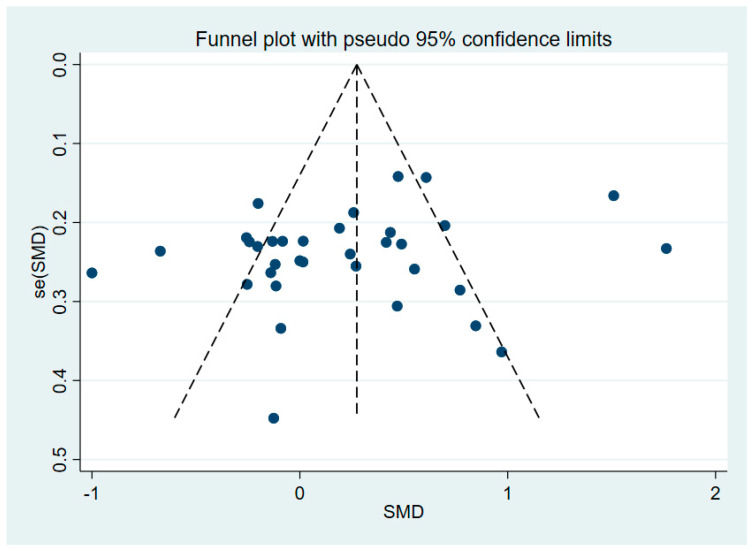
Funnel plot for HGS.

Stata16.0 software was used to construct a funnel plot of the HGS results (Figure 2), and Egger’s test was further employed to assess publication bias risk. It showed satisfactory symmetry between the left and right sides, and there was no significant publication bias (*p* = 0.207).

### 3.3. Outcomes

#### 3.3.1. Muscle Mass

The muscle mass indexes included in this study were BMI, FFM, SMM, and ASMM. A meta-analysis of seven studies comparing BMI between the intervention and control groups did not show significant differences. There was significant heterogeneity between the studies (*I*^2^ = 71%, *p* < 0.0005). When sensitivity analysis was performed, the exclusion of the study [33] led to a decrease in study heterogeneity (*I*^2^ = 60%), and the overall results also changed (MD: 0.32; 95% CI: 0.05, 0.59; *p* = 0.02). Heterogeneity decreased after the deletion of this article [33], which might be attributed to its small sample size.

Ten studies compared the FFM of the intervention group (nutritional supplement) with that of the control group (placebo). The meta-analysis results of these studies showed a significant difference in FFM between the two groups (MD: 0.72; 95% CI: 0.56, 0.87; *p* < 0.00001); FFM in the nutritional supplement group was significantly higher than that in the placebo group. There was significant heterogeneity between the studies (*I*^2^ = 86%, *p* < 0.00001). Although the heterogeneity between the studies did not significantly decrease after excluding each study one by one in the sensitivity analysis, subsequent subgroup analyses may have reduced heterogeneity. 

When comparing the SMM between the intervention and control groups, the results of the five studies showed no significant difference between the two groups (MD: 0.03; 95% CI: −0.20, 0.26; *p* = 0.78), and the heterogeneity between studies was not significant (*I*^2^ = 19%, *p* = 0.29).

**Figure 3:**
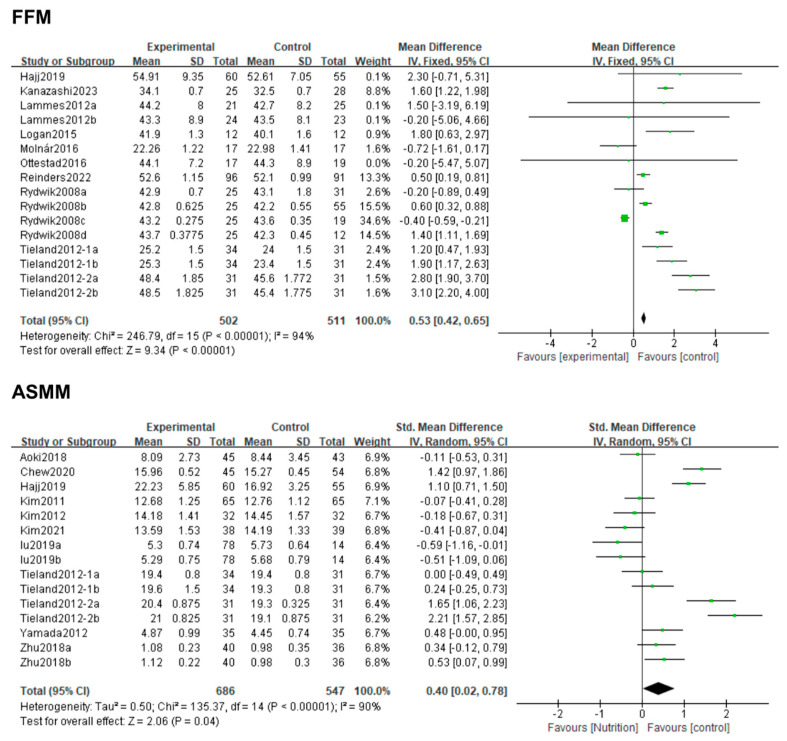
Forest plot for the effect of nutrition on FFM and ASMM. Note: Lammes 2012a [31]: Nutrition vs. control; Lammes 2012b [31]: Nutrition + resistance exercise vs. resistance exercise; Rydwik 2008a [40]: Nutrition vs. control, 12-week intervention; Rydwik 2008b [40]: Nutrition + resistance exercise vs. resistance exercise, 12-week intervention; Rydwik2008c [40]: Nutrition vs. control, 24-week intervention; Rydwik 2008d [40]: Nutrition + resistance exercise vs. resistance exercise, 24-week intervention; Tieland 2012-1a [41]: 12-week intervention; Tieland 2012-1b [41]: 24-week intervention; Tieland 2012-2a [42]: 12-week intervention; Tieland 2012-2b [42]: 12-week intervention; Zhu 2018a [11]: 12-week intervention; Zhu 2018b [11]: 24-week intervention.

Fifteen studies compared ASMM between the two groups, and significant differences (SMD: 0.40; 95% CI: 0.02, 0.78; *p* = 0.04) were noted between the intervention and control groups; the impact of nutritional supplements on ASMM was significantly higher in the intervention group compared to the control group. The heterogeneity (*I*^2^ = 90%, *p* < 0.00001) between the studies did not reveal a significant decrease after excluding each piece of literature one by one; nonetheless, subgroup analysis was still required (Figure 3).

#### 3.3.2. Muscle Strength

**Figure 4:**
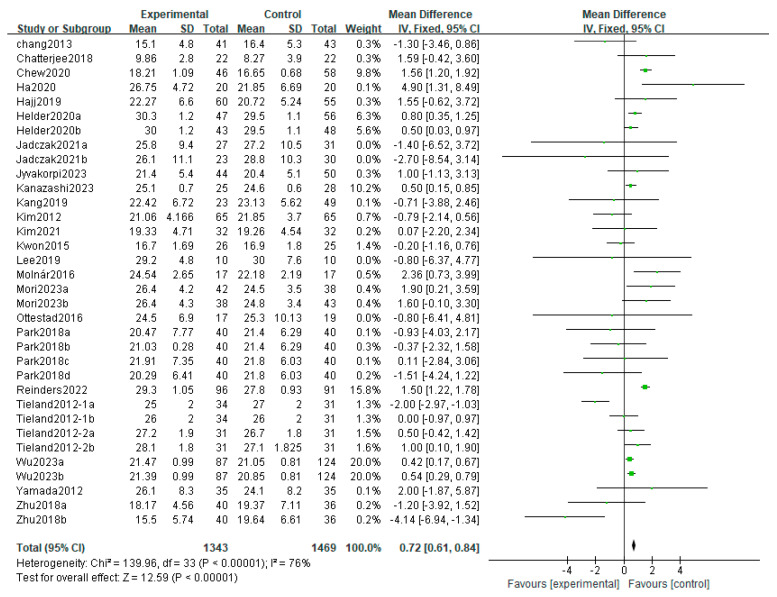
Forest plot for the effect of nutrition on HGS. Note: Jadczak2021a [24]: 12-week intervention; Jadczak2021b [24]: 24-week intervention; Helder2020a [9]: 12-week intervention; Helder2020b [9]: 12-week intervention; Mori2023a [36]: 12-week intervention; Mori2023b [36]: 24-week intervention; Park2018a [8]: protein 1.2 g/day, 6-week intervention; Park2018b [8]: protein 1.5 g/day, 6-week intervention; Park2018c [8]: protein 1.2 g/day, 12-week intervention; Park2018d [8]: protein 1.5 g/day, 12-week intervention; Wu2023a [43]: 12-week intervention; Wu2023b [43]: 24-week intervention.

Grip strength or handgrip strength (HGS) and strength of knee extension (SKE) are usually utilized as muscle strength test indicators. Significant differences in HGS (MD: 0.72; 95% CI: 0.61, 0.84; *p* < 0.00001) were found following the meta-analysis of 34 studies comparing HGS between the intervention and control groups. Moreover, HGS was significantly enhanced in the nutritional supplement groups (Figure 4). Moreover, there was a significant difference (MD: 0.64; 95% CI: 0.18, 1.11; *p* = 0.007) and heterogeneity (*I*^2^ = 42%, *p* = 0.06) in studies comparing the SKE between the nutritional supplement and placebo groups. 

#### 3.3.3. Physical Performance

**Figure 5:**
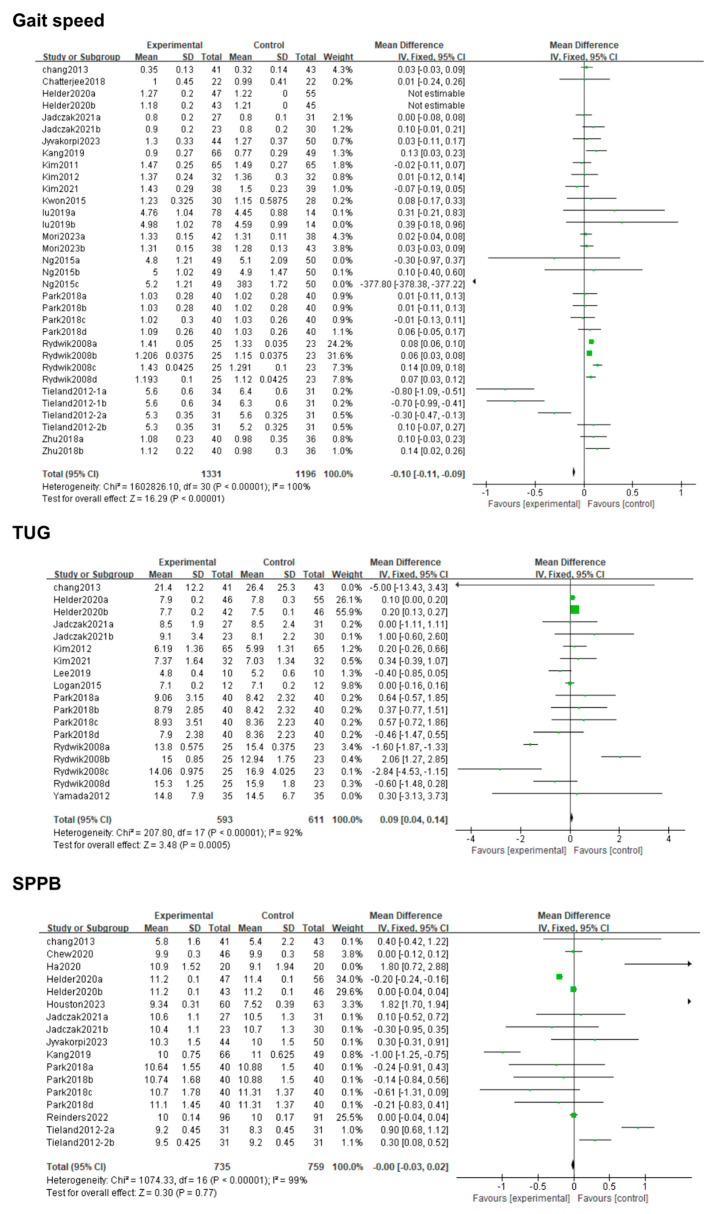
Forest plot for the effect of nutrition on gait speed, TUG, and SPPB. Note: Mori2023a [36]: 12-week intervention; Mori2023b [36]: 24-week intervention; Ng 2015a [6]: 12-week intervention; Ng 2015b [6]: 24-week intervention; Ng 2015c [6]: 48-week intervention.

The physical performance outcomes included gait speed, TUG, SPPB score, and PF. On the one hand, sixteen studies compared gait speed between an intervention group and a control group; the results showed that gait speed was significantly improved in the nutritional intervention group (MD: 006; 95% CI: 0.05, 0.07; *p* < 0.00001). On the other hand, eighteen studies compared TUG between the two groups, and the results uncovered that TUG was significantly prolonged in the intervention group (MD: 0.09; 95% CI: 0.04, 0.14; *p* = 0.005). Nevertheless, significant heterogeneity existed among the studies (*I*^2^ = 92%, *p* < 0.00001), which subsequently decreased (*I*^2^ = 27%) following the exclusion of the Rydwik 2008 study, which might be attributed to the nutritional types and doses used in the interventions. Seventeen studies compared the SPPB scores between the intervention and control groups (MD: −0.00; 95% CI: −0.03, 0.02; *p =* 0.77) (Figure 5). However, the results showed that the effect of nutritional supplements on the SPPB scores was significantly superior in the intervention group compared to the control group when excluding the Houston 2023 study (MD: −0.07; 95% CI: −0.09, −0.04; *p* < 0.00001). This may be due to the fact that the results of this paper did not change significantly, and the sample size was relatively large. Moreover, the heterogeneity among the studies (*I*^2^ = 99%, *p* < 0.00001) did not significantly decrease after excluding each study one by one. Therefore, subgroup analysis may be needed. Finally, four studies compared PF between the intervention and control groups; no significant difference in PF (MD: −0.10; 95% CI: −0.31, 0.12; *p* = 0.37) was found between the groups. However, heterogeneity was significant (*I*^2^ = 0%, *p* = 0.57) among the studies. 

### 3.4. Subgroup Analysis

#### 3.4.1. Nutritional Supplement Types

The results of subgroup analysis of nutritional supplement types on FFM, ASMM, HGS, gait speed, and TUG are shown in Table 4.

Herein, the administered nutritional interventions were protein, vitamin D, fish oil, energy (mainly energy supplements combined with other nutrients), and nutritional education. Owing to other types of nutritional interventions in the eligible studies, such as vitamin D [22], energy [37], and fish oil supplements [33], this study merely conducted a comparative subgroup analysis of FFM outcomes in the protein supplement and nutritional education groups. The subgroup analysis revealed that protein supplements had a superior interventional effect on FFM in the elderly (MD = 2.09, *p* < 0.0001). 

There were several studies on amino acid supplements, fish oil supplements, and nutritional education as nutritional interventions. Thus, only a subgroup analysis of protein, vitamin D, and energy supplements was conducted. The results of subgroup analysis demonstrated that in the elderly, protein supplement (SMD = 0.82, *p* < 0.0001) had the most excellent effect on ASMM, while vitamin D supplementation had a modest effect (SMD = 0.52, *p* < 0.0001), and energy supplementation had the lowest effect (SMD = 0.39, *p* = 0.0005).

In the HGS outcome analysis, a subgroup analysis of protein and energy supplements was carried out. The results showed that energy supplementation was effective in improving HGS (MD = 1.40, *p* < 0.0001).

Furthermore, the results of subgroup analysis showed that nutritional education (MD = 0.07, *p* < 0.0001) and the energy supplement (MD = 0.06, *p* = 0.01) had a positive effect on gait speed, but the effect was weak. 

Furthermore, significant differences were observed in the interventional effects between protein supplements and nutritional education on TUG. The results of subgroup analysis showed that the TUG was prolonged in the protein supplement group (MD = 0.21, *p* < 0.0001) and was significantly reduced by nutritional education (MD = −1.63, *p* < 0.0001). 

#### 3.4.2. Subject Age and Older Populations

A subgroup analysis was conducted, and the subjects were stratified by age (65–75 years and ≥75 years) and health status (healthy, frail, and sarcopenic). The results showed that the effect of nutritional intervention on the HGS (MD = 1.06, *p* < 0.0001) and TUG (MD = 0.14, *p* < 0.0001) of 65–75-year-old subjects was superior to that of subjects aged 75 years or older, while the nutritional intervention effect of ASMM (SMD = 0.61, *p* < 0.0001) was greater in individuals aged 75 years or older (Table 5).

The results of the older populations subgroup analysis demonstrated significant differences in the effects of nutritional intervention on outcomes among healthy, frail, and sarcopenic older adults (Table 6). Nutritional intervention had more significant effects on FFM (MD = 1.62, *p* < 0.0001) and HGS (MD = 0.82, *p* < 0.0001) in the frail and sarcopenic elderly individuals compared to healthy ones as well as a greater effect on ASMM (SMD = 0.69, *p* < 0.0001) in healthy and frail elderly subjects compared to sarcopenic ones. However, an adverse effect was observed in the TUG of the frail elderly subjects. Lastly, the gait speed of healthy elderly individuals was significantly improved (SMD = 0.08, *p* < 0.0001).

## 4. Discussion

### 4.1. Major Findings

The results in this meta-analysis show that nutritional supplementation improved FFM and ASMM and enhanced HGS and other muscle strength outcomes. Moreover, the positive effect of nutritional supplements on gait speed was statistically significant, with only a minor effect on this outcome. Nevertheless, nutritional supplements could not improve BMI, SMM, and SKE and might have negatively correlated with the TUG and SPPB scores. The included eligible studies were reviewed, yet a plausible explanation of the effect of nutritional intervention on muscle mass and physical performance in the elderly could not be established. This could be due to major limitations and inconsistencies in the study design or the overall low quality of the included studies.

### 4.2. The Effects of Nutritional Supplement Types

When evaluating studies of dietary supplements exhibiting significant effects, the heterogeneity of these studies was found to be very high. A large part of the source of this heterogeneity included factors such as the type of nutritional supplement products used, age, and so on. Therefore, subgroup analyses were conducted for each outcome indicator with significant differences. 

Previous reviews have corroborated that only essential amino acids and β-hydroxy β-methyl butyrate supplements exhibited consistent improvements in muscle mass and body performance parameters; protein supplementation had no consistent benefits in muscle mass, muscle strength, and body function [45]. Interestingly, the results of this systematic review are inconsistent with previous reviews, suggesting that protein supplements positively promoted FFM and ASMM outcomes related to muscle function and further improved FFM and ASMM compared to other nutritional supplements in the elderly. These findings could be attributed to differences in protein types or protein doses (ranging from 20 g/day to 45 g/day) in different studies. 

There are several theories explaining the mechanism by which proteins improve muscle. It was previously established that SMM is governed by the balance between muscle protein synthesis (MPS) and muscle protein breakdown (MPB), and the improvement in SMM is due to an MPS rate higher than the MPB rate [46]. Moreover, a vast body of evidence has shown that the potential of proteins to stimulate muscle protein synthesis is primarily dependent on their leucine content, which is a key signal to activate muscle protein synthesis and provide a substrate for the synthesis of new muscle proteins [47]. This study showed that protein supplements ameliorated SMM; therefore, we speculate that most of the proteins used in the eligible studies were derived from animal proteins [10,24,35]. Animal-derived proteins might be considered more anabolic than plant-based proteins owing to the abundance of leucine in animal proteins [47]. In terms of protein dose intake, total dietary protein and animal protein intake were associated with increased FFM in the elderly, according to data from the American Health, Aging and Body Composition Study. Earlier studies have established that a higher intake of plant proteins at each meal can result in a higher intake of essential amino acids (especially leucine), thus compensating for the lower muscle anabolic characteristics of plant and animal proteins [48]. Additionally, other studies have highlighted that protein intake less than 20 g/d has a more significant impact on FFM when considering the effect of protein on muscle mass, whereas a protein dose higher than 20 g/d has a greater impact on ASMM [49]. However, the analysis and discussion on protein types and amounts could not be expanded owing to the limited number of included studies.

This study also found inconsistent beneficial effects observed with some specific nutritional supplements, yet the results involved only one aspect of the muscle. For example, vitamin D and energy supplementations also increased the ASMM of the elderly in communities, but the effect of energy supplementation was lower than that of vitamin D supplementation. The interventional effect of energy supplements on HGS was significantly greater than that of protein supplements. Nutritional education not only significantly reduced the TUG of the elderly but also improved their gait speed by 0.07 m/s, thereby resulting in a significantly positive effect on physical performance. Although these supplements enhanced muscle function and physical activity, the effects on other indicators were inconsistent. Previous studies have reported that at the molecular level, impaired activation of the mechanistic target of rapamycin complex 1 (mTORC1) and downstream signals that regulate muscle protein synthesis (p70S6 kinase, 4E-BP1) might lead to anabolic resistance to aging [50,51]. It is hypothesized that this could be related to the anabolic resistance of the elderly to aging. Therefore, our results deemed that vitamin D, energy, and nutritional education had limited effects on partial muscle function in community seniors, and we hope that further studies on various types of nutritional interventions will be conducted in the future.

### 4.3. Effects of Age in Older Populations

The results of this study showed that nutritional intervention had a superior effect on HGS and TUG in individuals aged 65–75 years old compared to those aged 75 years or older, while the promotion of nutritional intervention on ASMM in older people aged 75 years or older was greater than that in 65–75-year-old subjects. Similarly, experimental studies conducted in Japan regarding the relationship between dietary protein intake, SMM, and HGS support the findings of this study. It was found that the correlation between dietary animal protein intake, ASMM, and MM differed among older Japanese women of different ages [15]. These results signal that dietary interventions alone might be less effective in preventing age-related muscle mass loss for the elderly aged 65–74 years, but interventions for muscle strength and physical activity might be appropriate and moderately effective. Another study on healthy individuals pointed out that the rate of muscle protein synthesis in older women was significantly higher than in men of a similar age [52]. Additionally, the enhancement effect of protein intake on muscle function was significantly different between genders [53], and protein intake was positively correlated with ASMM and MM in females but not in males [15]. These studies have validated that nutritional interventions have varying effects on older people of different ages and genders. 

There were significant differences in the improvement effects of nutritional interventions in different elderly populations. Nutritional intervention had the most pronounced effect on FFM, HGS, and ASMM in the frail elderly compared to healthy or sarcopenic elderly. Furthermore, with nutritional intervention, improvements in FFM and HGS in the frail elderly were greater than in the healthy elderly, while improvement in ASMM in the frail elderly was higher than in the sarcopenic elderly individuals. Previous studies concluded that nutritional interventions had greater effects on FFM in frail elderly [49] and demonstrated that nutritional supplementation had a marginal effect in elderly subjects with sarcopenia, implying that nutritional intervention alone did not have a significant effect in boosting muscle function. It is generally accepted that nutrition combined with exercise intervention might make it easier to achieve greater benefits in stimulating muscle mass and physical performance in sarcopenic patients [54]. Therefore, nutritional interventions might hold more promise in maintaining independence and preventing the decline in SMM and HGS in frail elderly people. The results of the current meta-analysis support the urgent need for further studies of nutritional interventions aimed at improving muscle function in elderly people who are frail or at risk of frailty in the community to prevent declines in muscle and physical activity function, especially in people at high risk of inadequate nutritional intake.

### 4.4. Advantages and Limitations

This study systematically evaluated the effects of several nutritional interventions on muscle health. It integrated the nutritional intervention methods and effects to promote muscle function of older people from the community. The results of this study might be conducive to the design of new and more advisable research as well as to encourage the exploration of more specific interventions, including nutritional support or combination approaches. However, there are some limitations to this study that need to be considered. First, the authors of the publications were contacted when the data were incomplete, but some might have been missed, although this is unlikely. Secondly, the importance of nutritional supplementation was systematically analyzed, but an intervention using physical exercise alone was not included, considering that this effect had been clarified in a recent meta-analysis. Future studies should incorporate larger sample sizes, standardized outcome measurements, and more detailed nutritional supplementation regimens in order to shed light on the benefits of nutritional interventions on muscle function in community-dwelling older people.

## 5. Conclusions

This study demonstrates that nutritional interventions are favorable to the muscle mass and physical performance of community-dwelling elderly individuals, but the optimal nutritional regimen to improve physical function and functioning is unclear. Regarding nutritional supplement selection, protein is recommended, considering it tends to have optimal effects on muscle function. Moreover, nutritional interventions had a superior effect on HGS and TUG in 65–75-year-old individuals and improved ASMM indicators in those aged 75 years or older. Therefore, we believe that to improve muscle function in the elderly, the golden period for implementing nutritional interventions is before 75 years. Regarding different older populations, nutritional interventions may be more promising in preventing the decline of FFM, HGS, and ASMM in frail elderly. Future studies should explore more extensive, detailed, and scientific nutrition programs that incorporate these characteristics to manage the health of older adults in communities.

## Figures and Tables

**Table 1:** Characteristics of included studies.

Reference	Study Location	Subjects	Study Design	Intervention Group	Control Group	Outcomes
Aoki, 2018 [18]	Japan (Asia)	IG: n = 43 (men: 11, woman: 32), age = 68.8 ± 5.3 CG: n = 45 (men: 11, woman: 34), age = 71.2 ± 6.8	24 weeks, 2 groups	(i) Ex + vitamin D group: Single-leg standing with opened eyes (1 min, 3 times a day) + squatting (five or six squats); commercial native vitamin D3, 25 mcg (1000 IU)·d^–1^.	(ii) Ex group: Single-leg standing with opened eyes (1 min, 3 times a day) + squatting (five or six squats).	ASMM
Chew, 2021 [19]	Singapore (Asia)	IG: n = 401 (men: 171, woman: 230), age = 74.3 ± 0.4 CG: n = 404 (men: 149, woman: 255), age = 74.0 ± 0.4	24 weeks, 2 groups	(i) Dietary counseling group: Beta-hydroxy-beta-methyl butyrate: 262 kcal, 10.5 g protein, 8.5 g fat, 34.2 g carbohydrate, 7.75 mcg (310 IU) vitamin D3, and 0.74 g calcium HMB per serving, 2 servings a day.	(ii) Placebo group: 60 kcal, 1.07 g protein, 1.21 g fat, and 11.9 g carbohydrate per serving, 2 servings a day.	HGS; SPPB; ASMM
Chatterjee, 2018 [20]	India (Asia)	IG: n = 22 (men: 13, woman: 9), age = 75.7 ± 6.3 CG: n = 22 (men: 18, woman: 4), age = 77.4 ± 3.8	12 weeks, 2 groups	(i) NW + INS (adapted indoor Nordic walking training +individualized nutritional supplementation): 60 min; supplementation providing additional 100 kcal with 8–10 g protein, protein-supplemented rate: 1.2 g·d^–1^.	(ii) NW: Adapted indoor Nordic walking training, 60 min.	Gait Speed; HGS
Ha, 2020 [21]	Korea (Asia)	IG: n = 22 (men: 6, woman: 16), age = 77.1 ± 6.4 CG: n = 22 (men: 10, woman: 12), age = 79.3 ± 4.9	12 weeks, 2 groups	(i) Ex +N group: 2–6 types of upper and lower-limb resistance exercises; nutritional and psychosocial interventions: 10–20 min, twice a week.	(ii) HE (health education): Lecture-based education 60 min, once a week.	HGS; SPPB
Hajj, 2019 [22]	Philippines (Asia)	IG: n = 60 (men: 32, woman: 27), age = 73.0 ± 2.0 CG: n = 55 (men: 26, woman: 29), age = 73.6 ± 2.1	24 weeks, 2 groups	(i) Vitamin D group: Cholecalciferol (Euro-Pharm International, Canada): vitamin D, three times a week.	(ii) Placebo group: a placebo tablet, three times a week.	HGS; ASMM; BMI; FFM
Helder, 2020 [9]	The Netherlands (Europe)	IG: n = 65 (men: 22, woman: 43), age = 72.3 ± 6.8 CG: n = 68 (men: 19, woman: 49), age = 70.8 ± 5.8	48 weeks, 2 groups	(i) HBex-Pro group: Blended home-based exercise training: weekly exercise. (ii) Nutrition intervention: Protein intake: minimum of 1.2 g·kg^−1^·d^–1^ and optimum of 1.5 g·kg^−1^·d^–1^.	(ii) HBex group: Community-based exercise program.	SPPB; TUG; Gait speed; HGS
Houston, 2023 [23]	Winston Salem (North America)	IG: n = 66 (men: 34, woman: 32), age = 73.7 ± 3.6 CG: n = 70 (men: 35, woman: 35), age = 73.1 ± 6.3	48 weeks, 2 groups	(i) Vitamin D group: 2000 IU/d vitamin D3.	(ii) Placebo group: placebo capsules (2000 IU/d).	SPPB
Jadczak, 2021 [24]	South Australia (Oceania)	IG: n = 34 (men: 10, woman: 24), age = 73.5 ± 7.2 CG: n = 36 (men: 13, woman: 23), age = 73.2 ± 6.6	24 weeks, 2 groups	(i) Whey Pro group: A powder in individual sachets containing 26 g of powder to provide 20 g of protein (essential amino acid 3 g and 11 g leucine), twice a day; supervised center-based group exercise class (60 min) + home based exercise sessions (strength, aerobic, balance, and flexibility tasks, 45 min) + walking (>30 min), twice a week.	(ii) RicePro group: Essential amino acid 2 g and 9 g leucine; isocaloric, isonitrogenous, and of comparable flavor and aroma.	HGS; Gait speed; SPPB; TUG; SMM; FFM
Jyvakorpi, 2023 [25]	Finland (Europe)	IG: n = 44, age = 75.0 ± 4.0 CG: n = 50, age = 76.0 ± 4.0	12 weeks, 2 groups	(i) Ex + N group: A simple five-movement exercise routine; a daily snack product containing MFGM and protein f.	(ii) Ex group: A simple five-movement exercise routine.	HGS; SPPB; Gait speed
Kanazashi, 2023 [26]	Japan (Asia)	IG: n = 25, age = 75 (72–78) CG: n = 28, age = 75 (69–78)	12 weeks, 2 groups	(i) Propolis group: 3 capsules (227 mg) of propolis, twice a day (after breakfast and dinner).	(ii) Placebo group: 3 capsules (227 mg) of placebo, twice a day (after breakfast and dinner).	BMI; FFM; HGS; SKE
Kang, 2019 [10]	China (Asia)	IG: n = 66 (men: 25, woman: 41), age = 76.8 ± 7.1 CG: n = 49 (men: 19, woman: 30), age = 78.0 ± 6.8	12 weeks, 2 groups	(i) Active group: RT + whey protein, 32.4 g·d^–1^, before breakfast and lunch or 30 min after resistance exercise, 30 min, twice a day.	(ii) Control group: RT, 30 min, twice a day.	HGS; Gait speed; SPPB; BMI
Kim, 2021 [7]	Japan (Asia)	IG: n = 65 (men: 0, woman: 65), age = 74.9 ± 5.4 CG: n = 65 (men: 0, woman: 65), age = 74.6 ± 4.9	12 weeks, 2 groups	(i). Ex + amino acid group: muscle-strengthening (30 min) + gait training (30 min, moderate intensity), twice a week; essential amino acid: 3 g leucine enriched essential amino acids (1.2 g leucine, 0.5 g lysine HCl, 0.33 g valine, 0.32 g isoleucine, 0.28 g threonine, 0.2 g phenylalanine, and 0.17 g others).	(ii) Ex + placebo group: Home-based exercise program: 29 min a day, 4.6 days/week; pills of a similar shape, taste, and texture as the amino-acid pills.	ASMM; SMI; HGS; TUG; BMI
Kim-Chang, 2013 [27]	Korea (Asia)	IG: n = 41, age = 78.9 ± 5.5 CG: n = 43, age = 78.4 ± 6.0	12 weeks, 2 groups	(i) N group: 200 mL of commercial liquid formula (400 kcal of energy, 25 g of protein, 9.4 g of essential amino acids, 56 g of carbohydrate, 9 g of lipid, 400 mL of water, and micronutrients), twice a day.	(ii) Control group: No treatments or counseling during the study period.	SPPB; Gait speed; TUG; HGS; PF
Kim, 2013 [28]	Japan (Asia)	IG: n = 32 (woman: 32), age = 80.6 ± 3.9 CG: n = 32 (woman: 32), age = 79.9 ± 4.9	12 weeks, 4 groups	(i) TC (tea catechin): Tea fortified, 350 mL (540 mg catechin), once a day (ii) Ex + TC: Muscle strength training (30 min) + gait and balance training (20 min), twice a week; tea fortified, 350 mL (540 mg catechin), once a day.	(iii) Ex: Muscle strength training (30 min) + gait and balance training. (iv) HE (health education): cognitive function, osteoporosis, and oral hygiene, once a month.	SMM; ASMM; HGS; TUG; Gait speed
Kim, 2012 [29]	Japan (Asia)	IG: n = 38 (woman: 38), age = 79.4 ± 2.8 CG: n = 39 (woman: 39), age = 78.9 ± 2.8	12 weeks, 4 groups	(i) Ex + AAS (amino acid supplements): A comprehensive physical fitness and muscle mass enhancement training program: 60 min, twice a week, moderate intensity; packets of powdered AAS, 3 g, twice a day. (ii) AAS: 3 g supplement, twice a day.	(iii) Exercise: Physical fitness and muscle training. (iv) HE: The classes focused on cognitive function, osteoporosis, and oral hygiene, once a month.	SMM; ASMM; BMI; Gait speed
Kwon, 2015 [30]	Japan (Asia)	IG: n = 30 (woman: 26), age = 77 ± 4.2 CG: n = 28 (woman: 25), age = 76.5 ± 3.8	12 weeks, 4 groups	(i) Ex+ N group: Stretching exercise (10–15 min) + muscle strength and balance capability (20–45 min), once a week; nutrition cooking class: 2–3 h, main ingredients used in the class were foods rich in protein and vitamin D.	(ii) Ex group: Stretching exercise (10–15 min) + muscle strength and balance (20–45 min), once a week.	HGS; Gait speed; PF
Lammes, 2012 [31]	Sweden (Europe)	IG: n = 49 (men: 37, woman: 12), age = 82.5 ± 4.2 CG: n = 44 (men: 36, woman: 8), age = 82.7 ± 2.7	12 weeks, 2 groups	(i) N + T group: endurance + muscle strength + balance, 60 min, twice a week; nutritional needs for the elderly, meal frequency, and cooking methods, 60 min.	(ii) T group: Endurance + muscle strength + balance, 60 min, twice a week.	FFM
Lee, 2019 [32]	New Mexico (North America)	IG: n = 10 (men: 3, woman: 7), age = 66.6 ± 4.4 CG: n = 10 (men: 4, woman: 6), age = 67.1 ± 7.3	12 weeks, 2 groups	(i) Fish oil + RT group: exercise training in the upper and lower body, 30 min, twice a week; Fish oil (eicosapentaenoic acid: 0.7 g, docosahexaenoic acid 0.24 g), three capsules a day.	(ii) RT group: RT: 30 min, twice a week; safflower oil: three capsules a day.	HGS; TUG
Logan, 2015 [33]	Britain (Europe)	IG: n = 12 (woman: 12), age = 66.0 ± 1.0 CG: n = 12 (woman: 12), age = 66.0 ± 1.0	12 weeks, 2 groups	(i) Fish oil group: Fish oil, five capsules (each capsule providing 400 mg of EPA and 200 mg of DHA), 5 g·d^–1^.	(ii) Placebo group: Placebo.	BMI; FFM; HGS; TUG
Lu, 2019 [34]	Singapore (Asia)	IG: n = 78 (men: 37, woman: 41), age = 69.8 ± 4.3 CG: n = 14 (men: 13, woman: 1), age = 71 ± 6.7	24 weeks, 2 groups	(i) Lifestyle interventions group: Resistance and balance training + nutritional enhancement with a commercial oral nutrition supplement formula + cognitive training.	(ii) Control group: Standard care.	ASMM; Gait speed
Molnár, 2016 [35]	Hungary (Europe)	IG: n = 17, age = 66.6 ± 2.7 CG: n = 17, age = 66.4 ± 2.4	12 weeks, 2 groups	(i) RT + N group: RT + strengthening exercises (30 min), twice a week; nutritional supplement (20 g whey protein, 10 g essential amino acid mixture, 3 g total leucine, 9 g carbohydrates, 3 g fat, 800 IU vitamin D, and a mixture of vitamins, minerals, and fibers per serving), twice a day.	(ii) RT group: RT + strengthening exercises (30 min), twice a week; standard physiotherapy.	FFM; HGS; SPPB
Mori, 2023 [36]	Japan (Asia)	IG: n = 46, age = 70.4 ± 4.6 CG: n = 46, age = 70.2 ± 5.3	24 weeks, 2 groups	(i) APP (Alaska pollack protein) group: test meal (breakfast) of APP powder (5.1 g; 4.5 g protein from Alaska pollack and 0.6 g protein from other sources) dissolved in 120 mL hot water.	(ii) Whey protein control group: test meal (breakfast) of control powder (5.0 g; 4.5 g protein from whey and 0.5 g protein from other sources) dissolved in 120 mL hot water.	SMM; HGS; Gait speed
Ng, 2015 [6]	Singapore (Asia)	IG: n = 98, age = 69.8 ± 4.2 CG: n = 50, age = 70.1 ± 5.0	48 weeks, 2 groups	(i) N group: Commercial formula (iron and folate supplement, vitamin B6 and vitamin B12 supplement, and calcium and vitamin D supplement), daily.	(ii) Control group: Usual care comparison.	BMI; Gait speed
Ottestad, 2016 [37]	Norway (Europe)	IG: n = 17 (men: 5, woman: 12), age = 76.8 ± 6.2 CG: n = 19 (men: 7, woman: 12), age = 77.1 ± 4.7	12 weeks, 2 groups	(i) Protein group: Protein-enriched milk (5.1% protein, 4.9% carbohydrates, <0.1% fat, and approximately 174 kJ (41 kcal)/100 g): 2 × 0.4 L·d^–1^, protein: 2 × 20 g·d^–1^.	(ii) Placebo group: An isocaloric carbohydrate: 178 mg·100 g^−1^ calcium and 0.1% Titadioksid.	SMM; HGS; FFM
Park, 2018 [8]	Korea (Asia)	IG: n = 40 (men: 16, woman: 24), age = 77.1 ± 3.7 CG: n = 40 (men: 14, woman: 26), age = 76.8 ± 3.9	12 weeks, 2 groups	(i) Protein supplement group: 1.5 g protein·kg^–1^·d^–1^, an individually adjusted amount of protein powder to fulfill 1.5 g·kg^–1^·d^–1^.	(ii) Control group: Packs containing placebo, 0.8 g·kg^–1^·d^–1^.	SPPB; Gait speed; TUG; HGS
Payette, 2002 [38]	Canada (North America)	IG: n = 42 (men: 12, woman: 29), age = 81.6 ± 7.5 CG: n = 41 (men: 12, woman: 29), age = 78.6 ± 6.1	16 weeks, 2 groups	(i) Protein group: Protein energy liquid supplement, 235 mL a day.	(ii) Control group: Placebo.	PF
Reinders, 2022 [39]	The Netherlands (Europe)	IG: n = 96 (men: 46, woman: 50), age = 75.9 ± 5.0 CG: n = 91 (men: 41, woman: 50), age = 75.0 ± 4.4	24 weeks, 2 groups	(i) Protein group: Advice of increasing protein intake (≥1.2 g/kg (aBW)/d).	(ii) Control group: Advice of the habitual diet (protein intake < 1.0 g/kg adjusted body weight (aBW)/d).	HGS; SPPB; FFM
Rydwik, 2008 [40]	Sweden (Europe)	IG: n = 50 (men: 19, woman: 31), age = 83.1 ± 4.2 CG: n = 46 (men: 19, woman: 27), age = 83.2 ± 3.8	24 weeks, 4 groups	(i) T + N group: Aerobic training + muscle strength training (60–80% intensity) + Qigong, one hour, twice a week; individual dietary counseling, 1 h. (ii) N group: Individual dietary counseling.	(iii) T group: Aerobic+ muscle strength training + Qigong, 1 h. (iv) C group: Control nutrition and training.	TUG; FFM; TUG
Tieland, 2012-1 [41]	The Netherlands (Europe)	IG: n = 34 (men: 20, woman: 24), age = 78.0 ± 1.0 CG: n = 31 (men: 16, woman: 15), age = 81.0 ± 1.0	24 weeks, 2 groups	(i) Protein group: 250 mL beverage (15 g protein (milk protein concentrate, 7.1 g lactose, 0.5 g fat, and 0.4 g calcium), twice a day.	(ii) Placebo group: 250 mL beverage (no protein, 7.1 g lactose, 0.4 g calcium).	HGS; Gait speed
Tieland, 2012-2 [42]	The Netherlands (Europe)	IG: n = 31 (men: 25, woman: 6), age = 78.0 ± 9.0 CG: n = 31 (men: 24, woman: 7), age = 79.0 ± 6.0	24 weeks, 2 groups	(i) RT + Protein group: RT, 50% 1—RM (10–15 repetitions per set)—75% 1—RM, twice a week; protein supplementation (15 g protein, 7.1 g lactose, 0.5 g fat, and 0.4 g calcium), 250 mL, twice a day.	(ii) RT + Placebo group: RT, twice a week; placebo (no protein, 7.1 g lactose, and 0.4 g calcium), 250 mL, twice a day.	FFM; HGS; SPPB; Gait speed
Wu, 2023 [43]	Taiwan, China (Asia)	IG: n = 87(men: 24, woman: 63), age = 75.2 ± 0.7 CG: n = 124 (men: 26, woman: 98), age = 73.1 ± 0.6	24 weeks, 2 groups	(i) Ex + N group: Nutritional education and weekly one-hour exercises including aerobic exercise, muscle strength training, balance and coordination exercise, and stretching.	(ii) Ex group: weekly one-hour exercises including aerobic exercise, muscle strength training, balance and coordination exercise, and stretching.	HGS
Yamada, 2012 [44]	Japan (Asia)	IG: n = 35 (men: 21, woman: 17), age = 74.4 ± 7.3 CG: n = 35 (men: 20, woman: 19), age = 75.6 ± 6.0	12 weeks, 2 groups	(i) N + RT: RT (60 min) + flexibility and balance exercise (10 min), 3 times a week; a multi-nutrient supplementation (12.5 μg of vitamin D, 10 g of protein with branched-chain amino acids, 200 kcal, 41% carbohydrate, 37% fat, 20% protein, 2% oligosaccharide), three times a week.	(ii) RT group: RT (60 min) + flexibility and balance exercise (10 min), three times a week.	TUG; HGS; ASMM;
Zhu, 2018 [11]	Hong Kong, China (Asia)	IG: n = 40 (men: 11, woman: 29), age = 74.5 ± 6.9 CG: n = 36 (men: 7, woman: 29), age = 74.8 ± 7.1	24 weeks, 2 groups	(i) N + Ex: RT (20–30 min, chair-based resistance exercise) + AE (20 min), twice a week; nutrition supplement (231 calories, 8.61 g protein, 1.21 g β-hydroxyl β-methylbutyrate, 130 IU vitamin D and 0.29 g omega—3 fatty acid): 54.1 g powder, twice a day.	(ii) Ex group: RT (20–30 min, chair-based resistance exercise) + AE (20 min), twice a week.	Gait speed; HGS; ASMM

Note: IG, intervention group; CG, control group; Ex, exercise; RT, resistance training; AE, aerobic exercises; N, nutrition; T, training; BMI, body mass index; FFM, fat-free mass or lean mass; SMM, skeletal muscle mass; ASMM, appendicular skeletal muscle mass; HGS, grip strength or handgrip strength; SKE, strength of knee extension; TUG, timed up and go test; PF, physical function.

**Table 2:** Risk of bias assessment of included studies.

References	Random Sequence Generation	Allocation Concealment	Blinding of Participants and Personnel	Blinding of Outcome Assessment	Incomplete Outcome Data	Selective Reporting	Other Sources of Bias
Aoki, 2018 [18]	Low risk	Low risk	High risk	Unclear risk	Low risk	Unclear risk	Unclear risk
Chew, 2020 [19]	Low risk	Low risk	Unclear risk	Unclear risk	Unclear risk	Unclear risk	Unclear risk
Chatterjee, 2018 [20]	Low risk	Low risk	Unclear risk	Unclear risk	Low risk	Unclear risk	Unclear risk
Ha, 2020 [21]	Low risk	Low risk	Unclear risk	Unclear risk	Low risk	Unclear risk	Unclear risk
Hajj, 2019 [22]	Low risk	Low risk	Low risk	Unclear risk	Unclear risk	Unclear risk	Unclear risk
Helder, 2020 [9]	Unclear risk	Unclear risk	Unclear risk	Unclear risk	Low risk	Unclear risk	Unclear risk
Houston, 2023 [23]	Low risk	Low risk	Low risk	Low risk	Low risk	Low risk	Unclear risk
Jadczak, 2021 [24]	Low risk	Low risk	Unclear risk	Unclear risk	Low risk	Unclear risk	Unclear risk
Jyvakorpi, 2023 [25]	Unclear risk	Unclear risk	Unclear risk	Unclear risk	Low risk	Unclear risk	Unclear risk
Kanazashi, 2023 [26]	Low risk	Low risk	Low risk	Unclear risk	Low risk	Unclear risk	Unclear risk
Kang, 2019 [10]	Unclear risk	Unclear risk	Unclear risk	Unclear risk	Low risk	Unclear risk	Unclear risk
Kim, 2021 [7]	Low risk	Low risk	Low risk	Low risk	Low risk	Low risk	Unclear risk
Kim-Chang, 2013 [27]	Low risk	Low risk	Low risk	Low risk	Low risk	Low risk	Unclear risk
Kim, 2013 [28]	Low risk	Low risk	Low risk	Low risk	Low risk	Low risk	Unclear risk
Kim, 2012 [29]	Low risk	Low risk	Unclear risk	Unclear risk	Unclear risk	Unclear risk	Unclear risk
Kwon, 2015 [30]	Low risk	Low risk	Low risk	Low risk	Unclear risk	Unclear risk	Unclear risk
Lammes, 2012 [31]	Unclear risk	Unclear risk	Unclear risk	Unclear risk	Unclear risk	Unclear risk	Unclear risk
Lee, 2019 [32]	Unclear risk	Unclear risk	Unclear risk	Unclear risk	Low risk	Unclear risk	Unclear risk
Logan, 2015 [33]	Low risk	Unclear risk	Unclear risk	Unclear risk	Low risk	Unclear risk	Unclear risk
Lu, 2019 [34]	Low risk	Unclear risk	Unclear risk	Unclear risk	Low risk	Unclear risk	Unclear risk
Molnár, 2016 [35]	Unclear risk	Unclear risk	Unclear risk	Unclear risk	Low risk	Unclear risk	Unclear risk
Mori, 2023 [36]	Low risk	Unclear risk	Low risk	Unclear risk	Low risk	Low risk	Unclear risk
Ng, 2015 [6]	Low risk	Low risk	Low risk	Unclear risk	Low risk	Unclear risk	Unclear risk
Ottestad, 2016 [37]	Low risk	Low risk	Low risk	Low risk	Low risk	Low risk	Unclear risk
Park, 2018 [8]	Low risk	Low risk	Low risk	Low risk	Low risk	Low risk	Unclear risk
Payette, 2002 [38]	Low risk	Unclear risk	Low risk	Unclear risk	Unclear risk	Unclear risk	Unclear risk
Reinders, 2022 [39]	Low risk	Low risk	Unclear risk	Unclear risk	Low risk	Low risk	Unclear risk
Rydwik, 2008 [40]	Unclear risk	Unclear risk	Unclear risk	Unclear risk	Low risk	Unclear risk	Unclear risk
Tieland, 2012-1 [41]	Low risk	Low risk	Low risk	Unclear risk	Low risk	Unclear risk	Unclear risk
Tieland, 2012-2 [42]	Low risk	Unclear risk	Unclear risk	Unclear risk	Unclear risk	Unclear risk	Unclear risk
Wu, 2023 [43]	Low risk	Unclear risk	Unclear risk	Unclear risk	Low risk	Low risk	Unclear risk
Yamada, 2012 [44]	Unclear risk	Unclear risk	Unclear risk	Unclear risk	Low risk	Unclear risk	Unclear risk
Zhu, 2018 [11]	Low risk	Low risk	Low risk	Unclear risk	Low risk	Unclear risk	Unclear risk

**Table 3:** Certainty of evidence assessed using GRADE.

Quality Assessment							No of Patients		Effect		Certainty	Importance
No of Studies	Study Design	Risk of Bias	Inconsistency	Indirectness	Imprecision	Other Considerations	Nutritional Intervention	Control	Relative (95% CI)	Absolute (95% CI)		
SMM												
7	Randomized trials	Not serious	Not serious	Not serious	Not serious	None	255	269	-	MD 0.03 higher (0.2 lower to 0.26 higher)	⨁⨁⨁⨁ HIGH	IMPORTANT
Fat-free Mass												
16	Randomized trials	Not serious	Very serious ^a^	Not serious	Not serious	None	502	511	-	MD 0.53 higher (0.42 higher to 0.65 higher)	⨁⨁◯◯ LOW	IMPORTANT
BMI												
8	Randomized trials	Not serious	Serious ^b^	Not serious	Not serious	None	364	348	-	MD 0.32 higher (0.05 higher to 0.59 higher)	⨁⨁⨁◯ MODERATE	IMPORTANT
ASMM												
15	Randomized trials	Not serious	Very serious ^c^	Not serious	Not serious	None	686	547	-	SMD 0.4 higher (0.02 higher to 0.78 higher)	⨁⨁◯◯ LOW	CRITICAL
HGS												
34	Randomized trials	Not serious	Serious ^d^	Not serious	Not serious	None	1343	1469	-	MD 0.72 higher (0.61 higher to 0.84 higher)	⨁⨁⨁◯ MODERATE	CRITICAL
KS												
12	Randomized trials	Not serious	Not serious	Not serious	Not serious	None	497	500	-	MD 0.62 higher (0.17 higher to 1.08 higher)	⨁⨁⨁⨁ HIGH	IMPORTANT
SPPB												
17	Randomized trials	Not serious	Very serious ^e^	Not serious	Not serious	None	735	759	-	MD 0 lower (0.03 lower to 0.02 higher)	⨁⨁◯◯ LOW	CRITICAL
Physical Functioning												
5	Randomized trials	Not serious	Not serious	Not serious	Not serious	None	154	184	-	MD 0.06 lower (0.15 lower to 0.03 higher)	⨁⨁⨁⨁ HIGH	IMPORTANT
TUG												
18	Randomized trials	Not serious	Very serious ^f^	Not serious	Not serious	None	593	611	-	MD 0.09 higher (0.04 higher to 0.14 higher)	⨁⨁◯◯ LOW	CRITICAL
Gait Speed												
33	Randomized trials	Not serious	Very serious ^g^	Not serious	Not serious	None	1331	1196	-	MD 0.1 lower (0.11 lower to 0.09 lower)	⨁⨁◯◯ LOW	CRITICAL
Physical Activity Level												
13	Randomized trials	Not serious	Not serious	Not serious	Not serious	None	492	520	-	SMD 0.01 lower (0.14 lower to 0.13 higher)	⨁⨁⨁⨁ HIGH	IMPORTANT
PF												
4	Randomized trials	Not serious	Not serious	Not serious	Not serious	None	154	184	-	SMD 0.1 lower (0.31 lower to 0.12 higher)	⨁⨁⨁⨁ HIGH	IMPORTANT
Frail Score												
8	Randomized trials	Not serious	Not serious	Not serious	Not serious	None	312	322	-	MD 0.02 higher (0.13 lower to 0.16 higher)	⨁⨁⨁⨁ HIGH	IMPORTANT

^a^ The choice of intervention modality and the difference in physical condition (healthy or frail) have a large impact on FFM outcomes, and subgroup analysis is required. ^b^ The small sample size of one of the included studies [33] may have affected the heterogeneity results. ^c^ The choice of type of nutritional supplement and age can have a large impact on ASM results and therefore need to be analyzed in subgroups. ^d^ Physical status (healthy or frail), age, duration of intervention, and choice of nutritional supplements in older people all have an impact on HG outcomes and therefore need to be discussed in subgroups. ^e^ The age of the older person, the choice of intervention modality, and duration of intervention all have an impact on the SPPB results and therefore need to be discussed in subgroups. ^f^ Age, physical condition (healthy or debilitated), and choice of nutritional supplements in the elderly all have an impact on TUG results and therefore need to be discussed in subgroups. ^g^ The intervention modality, duration of intervention, and choice of nutritional supplements all have an impact on the gait speed outcomes and therefore need to be discussed in subgroups.

**Table 4:** Subgroup analysis of nutritional supplement types.

Subgroups	Subjects (n)	MD/SMD (95% CI)	*p*-Value of Overall Effect	*I*^2^ (%)
FFM (kg)				
Protein supplement	290	2.09 [1.69, 2.49]	*p* < 0.0001	71
Nutrition education	497	0.27 [0.15, 0.40]	*p* < 0.0001	95
ASMM (kg)				
Protein supplement	254	0.82 [0.55, 1.09]	*p* < 0.0001	93
Vitamin D	273	0.52 [0.27, 0.76]	*p* < 0.0001	88
Energy	435	0.39 [0.17, 0.61]	*p* = 0.0005	90
HGS (kg)				
Protein supplement	1242	0.43 [0.18, 0.68]	*p* = 0.0009	60
Energy	418	1.40 [1.06, 1.74]	*p* < 0.0001	81
		Gait speed (m/s)		
Protein supplement	1245	0.01 [−0.01, 0.04]	*p* = 0.30	81
Nutrition education	250	0.07 [0.06, 0.09]	*p* < 0.0001	65
Energy	761	0.06 [0.01, 0.11]	*p* = 0.01	0
		TUG (m/s)		
Protein supplement	620	0.21 [0.15, 0.26]	*p* < 0.0001	98
Nutrition education	96	−1.63 [−1.90, −1.36]	*p* < 0.0001	98

**Table 5:** Subgroup analysis of subject age.

Subgroups	Subjects (n)	MD/SMD (95% CI)	*p*-Value of Overall Effect	*I*^2^ (%)
ASMM (kg)				
65–75 years	715	0.17 [0.09, 0.25]	*p* < 0.0001	92
≥75 years	518	0.61 [0.44, 0.77]	*p* < 0.0001	92
HGS (kg)				
65–75 years	995	1.06 [0.83, 1.29]	*p* < 0.0001	69
≥75 years	1061	−0.16 [−0.52, 0.21]	*p* = 0.04	53
TUG (m/s)				
65–75 years	408	0.14 [0.09, 0.20]	*p* < 0.0001	56
≥75 years	796	−0.22 [−0.42, −0.02]	*p* = 0.03	98

**Table 6:** Subgroup analysis of older populations.

Subgroups	Subjects (n)	MD/SMD (95% CI)	*p*-Value of Overall Effect	*I*^2^ (%)
FFM (kg)				
Healthy	277	0.25 [0.11, 0.38]	*p* = 0.0003	96
Frail	381	1.62 [1.25, 1.98]	*p* < 0.0001	87
ASMM (kg)				
Frail	500	0.69 [0.56, 0.82]	*p* < 0.0001	91
Sarcopenic	645	0.08 [−0.01, 0.16]	*p* = 0.07	88
HGS (kg)				
Healthy	464	0.63 [0.39, 0.86]	*p* < 0.0001	0
Frail	1248	0.82 [0.56, 1.07]	*p* < 0.0001	77
Sarcopenic	397	−0.78 [−1.77, 0.21]	*p* = 0.12	70
Gait speed (m/s)				
Healthy	382	0.08 [0.06, 0.09]	*p* < 0.0001	69
Frail	1360	0.01 [−0.02, 0.04]	*p* = 0.72	78
Sarcopenic	530	0.05 [−0.01, 0.11]	*p* = 0.08	36
TUG (s)				
Healthy	405	0.09 [0.04, 0.14]	*p* = 0.0005	97
Frail	837	0.48 [0.17, 0.78]	*p* = 0.002	28

## Data Availability

Data are available upon request from the correspondence author.
